# Supplementary material for: Influence of cardiovascular risk factors and treatment exposure on cardiovascular event incidence: Assessment using machine learning algorithms
Source: PLoS One. 2023 Nov 16;18(11):e0293759. doi: 10.1371/journal.pone.0293759 (PMC10653526; doi:10.1371/journal.pone.0293759)
Supplement: Attachment — Submitted filename: Response to reviewers.docx [file pone.0293759.s001.docx]

**Reviewer #1: Thank you for the opportunity to read this fascinating article. Nonetheless, I would like to make a few suggestions.**

*We want to thank the reviewer her suggestions, which have been useful to improve the quality and clarity of the manuscript. We proceed to answer, point by point, his/her comments:*

**1. The introduction is extremely lengthy. Please trim it down, particularly lines 71 to 81. The authors could instead provide a reference to these well-known algorithms and, more importantly, examples of their use in CVD risk prediction.**

*Thank you for your comment. As suggested by the reviewer, we have reduced the introduction, and the paragraph suggested now is as follows (lines 73-76):*

*“There are different kinds of supervised machine learning. When the variable to be predicted is categorical (e.g. a cardiovascular event), classification machine learning techniques [3,4] such as Naïve Bayes (NB) algorithms and ensemble methods are used [12,14,15]. Ensemble methods include bagging and boosting methods such are Random Forest (RF), XG Boost [12,14,15]. “*

**2. Justify the algorithm selections for machine learning.**

*To justify the algorithms selected in the present study, we have added some references in which these algorithms are applied to predict cardiovascular disease (lines 73-76):*

*“When the variable to be predicted is categorical (e.g. a cardiovascular event), classification machine learning techniques [3,4] such as Naïve Bayes (NB) algorithms and ensemble methods are used [12,14,15]. Ensemble methods include bagging and boosting methods such are Random Forest (RF), XG Boost[12,14,15].”*

*References:*

*3. Alaa AM, Bolton T, Angelantonio E di, Rudd JHF, van der Schaar M. Cardiovascular disease risk prediction using automated machine learning: A prospective study of 423,604 UK Biobank participants. PLoS One [Internet]. 2019 May 1 [cited 2021 May 14];14(5). Available from:* [*https://pubmed.ncbi.nlm.nih.gov/31091238/*](https://pubmed.ncbi.nlm.nih.gov/31091238/)

*4. Ambale-Venkatesh B, Yang X, Wu CO, Liu K, Gregory Hundley W, McClelland R, et al. Cardiovascular Event Prediction by Machine Learning: The Multi-Ethnic Study of Atherosclerosis. Circ Res. 2017 Oct 13;121(9):1092–101.*

*12. Wang K, Tian J, Zheng C, Yang H, Ren J, Liu Y, et al. Interpretable prediction of 3-year all-cause mortality in patients with heart failure caused by coronary heart disease based on machine learning and SHAP. Comput Biol Med. 2021 Oct 1;137:104813.*

*14. Tsarapatsani K, Sakellarios AI, Pezoulas VC, Tsakanikas VD, Kleber ME, Marz W, et al. Machine Learning Models for Cardiovascular Disease Events Prediction. Annu Int Conf IEEE Eng Med Biol Soc [Internet]. 2022 [cited 2023 Sep 13];2022:1066–9. Available from:* [*https://pubmed.ncbi.nlm.nih.gov/36085658/*](https://pubmed.ncbi.nlm.nih.gov/36085658/)

*15. Garavand A, Salehnasab C, Behmanesh A, Aslani N, Zadeh AH, Ghaderzadeh M. Efficient Model for Coronary Artery Disease Diagnosis: A Comparative Study of Several Machine Learning Algorithms. J Healthc Eng. 2022.*

**3. Line 64 – 66 As LIME or SHAP analysis exists to overcome the black box nature of the machine learning algorithm, it appears that the authors' claims are irrelevant. Consider including either method in this study.**

*Thank you for your comment. We analyse the importance of features applying caret package in R^[1]^. To clarify it we added some lines in the introduction and in the methods as follows:*

*Introduction (lines 67-68):*

*“This problem has been overcome by the development of different methods, implemented in different R libraries, that try to set the importance of each feature in the prediction [10–12].*

*Methodology (lines 231-243):*

“*For RF models, the method applied to compute the contribution of each variable consisted of recoding for each tree the prediction accuracy on the out-of-bag portion. Then each predictor variable was permuted and the same was done. Finally, for all trees, the difference between both accuracies was averaged and normalized by the standard score [10,11].*

*For XG Boost models, the reduction in the loss function attributable to each variable in sum of squared error in predicting the gradient on each iteration was calculated. Finally, the improvement score for each predictor was averaged across all the trees in the ensemble [11,25].*

*Finally, for models developed applying NB a ROC curve analysis was conducted on each predictor. Different cutoffs were applied to the predictor data to predict the class. Then, sensitivity and specificity were computed for each cutoff and the area under ROC curve was calculated using the trapezoidal rule. This area was used as the measure of variable importance[11].”*

*References:*

*10.	Greenwell BM, Boehmke BC. Variable Importance Plots-An Introduction to the vip Package.*

*11.	Kuhn M. Building Predictive Models in R Using the caret Package. J Stat Softw [Internet]. 2008;28(5). Available from:* [*http://www.jstatsoft.org/*](http://www.jstatsoft.org/)

*12.	Wang K, Tian J, Zheng C, Yang H, Ren J, Liu Y, et al. Interpretable prediction of 3-year all-cause mortality in patients with heart failure caused by coronary heart disease based on machine learning and SHAP. Comput Biol Med. 2021 Oct 1;137:104813.*

*25.	Jiaming Yuan. Package ‘xgboost’. 2023 [cited 2023 Sep 14]; Available from: https://github.com/dmlc/xgboost/issues*

**4. SMOTE is one example of balancing the data up and down to achieve balance should consider using it.**

*Thank you for the comment. According to the literature^[2]^^[3]^^[4]^, there are different methods to deal with imbalanced data, in this study it was applied a threshold-moving and the parameter chosen to select the best threshold was the F1 score^[5]^. Further explanations and bibliography will be given in comment number 11.*

**5. Please refer, between lines 82 and 83, to papers that employ the Machine Learning algorithm for CVD risk estimation instead of other diseases.**

*As suggested, we added some examples of manuscripts that use machine learning techniques to predict cardiovascular disease (line 77-78):*

*“Machine learning techniques represent a promising approach for CVD risk prediction given their demonstrated potential [3,4,12,14–16] and the advantages they offer over existing scoring systems.”*

*References:*

*3.	Alaa AM, Bolton T, Angelantonio E Di, Rudd JHF, van der Schaar M. Cardiovascular disease risk prediction using automated machine learning: A prospective study of 423,604 UK Biobank participants. PLoS One [Internet]. 2019 May 1;14(5). Available from: https://pubmed.ncbi.nlm.nih.gov/31091238/*

*4.	Ambale-Venkatesh B, Yang X, Wu CO, Liu K, Gregory Hundley W, McClelland R, et al. Cardiovascular Event Prediction by Machine Learning: The Multi-Ethnic Study of Atherosclerosis. Circ Res. 2017 Oct 13;121(9):1092–101.*

*12.	Wang K, Tian J, Zheng C, Yang H, Ren J, Liu Y, et al. Interpretable prediction of 3-year all-cause mortality in patients with heart failure caused by coronary heart disease based on machine learning and SHAP. Comput Biol Med. 2021 Oct 1;137:104813.*

*14.	Tsarapatsani K, Sakellarios AI, Pezoulas VC, Tsakanikas VD, Kleber ME, Marz W, et al. Machine Learning Models for Cardiovascular Disease Events Prediction. Annu Int Conf IEEE Eng Med Biol Soc [Internet]. 2022 [cited 2023 Sep 13];2022:1066–9. Available from: https://pubmed.ncbi.nlm.nih.gov/36085658/*

*15.	Garavand A, Salehnasab C, Behmanesh A, Aslani N, Zadeh AH, Ghaderzadeh M. Efficient Model for Coronary Artery Disease Diagnosis: A Comparative Study of Several Machine Learning Algorithms. J Healthc Eng. 2022;2022.*

*16.	Aziz F, Malek S, Ibrahim KS, Shariff RER, Wan Ahmad WA, Ali RM, et al. Short- and long-term mortality prediction after an acute ST-elevation myocardial infarction (STEMI) in Asians: A machine learning approach. PLoS One [Internet]. 2021 Aug 1 [cited 2023 Sep 14];16(8):e0254894. Available from: https://journals.plos.org/plosone/article?id=10.1371/journal.pone.0254894*
 **6. Please rewrite the purpose of this study and specify which algorithm is being employed.**

*As suggested by the reviewer, we added the algorithms in the objectives (lines 78-81):*

*“In the present study, we analyzed the ability of three machine learning algorithms, NB, RF and XG Boost, to predict the appearance of cardiovascular events (CVE) and analysed the influence of different CVRF-related variables on CVE prediction.”*

**7. Please also consider including a comparison with the conventional FRS score. Please specify the limitation of the FRS. Has it been validated in your population? If so, what are the outcomes?**

*FRS was calibrated for a Spanish population^[6]^ and it has been validated in other Spanish cohorts showing different results^[7]^^[8]^^[9]^ . Nevertheless, one of the objectives of the present study was to analise the role of adherence to treatment in the prediction of CVE, and FRS score does not consider this information. Furthermore, some of the variables included in the FRS were not available during the years that the study was conducted as it is explained in the limitations as follows (line 394-397):*

*“Other limitations, related to the kind of data available, include the absence of smoking data for the entire study period, since smoking is one of the most important modifiable risk factors for CVE.”*

*Finally, we included a sentence about the comparison of machine learning techniques and FRS in the Discussion (lines 385-386) as follows:*

*“Finally, when comparing performance of RF and XGBoost algorithms with SCORE and Framingham risk score, the first ones had better results [5]. “*

*Reference:*

*5. Jamthikar AD, Gupta D, Mantella LE, Saba L, Laird JR, Johri AM, et al. Multiclass machine learning vs. conventional calculators for stroke/CVD risk assessment using carotid plaque predictors with coronary angiography scores as gold standard: a 500 participants study. Int J Cardiovasc Imaging. 2021 Apr 1;37(4):1171–87. doi:10.1007/S10554-020-02099-7/TABLES/5*

Methods
**8. Provide a flowchart and describe the total number of participants from various data sources. Include the percentage of missing data for each variable utilised in this study.**

*Thank you for your comment. To clarify the selection of participants and the model development procedure we introduced a flowchart in the methods section. To explain how variables were extracted, its origin and the number of missing data, we did a table and we introduced both of them in the methods section as follows:*

*Line 96: “The selection of patients is explained in Figure 1.”*

Fig 1: Flowchart depicting the study population and model development.

*Line 115:*

*The source of the variables and number of missing data is summarised in Table 1.*

Table 1: Variables sources and missing data

|  | **AWHS** | **BIGAN** | | | **General Direction of Public Health** |  |
| --- | --- | --- | --- | --- | --- | --- |
| **Variables** | **Annual medical test** | **Pharmaceutical dispensation database** | **Minimum Basic Data Set** | **Emergency Database** | **Aragon Mortality Registry** | **NA** |
| **Hypertension** | X | X |  |  |  | 26 (0.5%) |
| **Hypercholesterolemia** | X | X |  |  |  | 14 (0.3%) |
| **Diabetes** | X | X |  |  |  | 27 (0.6%) |
| **Physical status** | X |  |  |  |  | 57 (1.2%) |
| **Age** | X |  |  |  |  | 0 |
| **Treatment exposure** |  | X |  |  |  | 0 |
| **CVE** |  |  | X | X | X | 0 |

**9. Since data is randomly divided, are there duplicate records for the same patients?**

*Thank you for your comment. It was applied a random sampling without replacement, so there were not duplicate records for any patient.*

**10. Line 198 – 206 The authors' claims that the data is separated into training and testing data and that the entire data set is also used for testing are unclear. If the number of datasets is low, it is recommended to use K fold.**

*As it is explained in lines 202-205, 5 fold and 10 fold stratification was applied to train models and to adjust the parameters in the different models. To testing the models all the sample was considered.*

*“To fit the hyperparameters and avoid over-fitting, the prediction accuracy of all models was tested using 5 and 10 fold stratified cross validation (cv) to estimate F1-score. Results were similar when applying 5 and 10 fold cv, so results shown in the article corresponds to the 5-fold cv.”*

**11. Moreover, what measures are taken to address data imbalances? Is it rational to change the threshold value to 0.1? What scientific evidence supports this claim? Provide references for justifications of any steps.**

*Thank you for your comment. To address data imbalanced we have applied a threshold-moving attending to the F1-measure. After analyzing different thresholds to evaluate which maximizes F1-measure, it pointed to 0.1 as the best threshold in all the models computed. We added a section in the methodology to explain how the models assessment was done and added some references (lines 219-226):*

*“Machine learning models assessment*

*Because of the highly imbalanced data that we had, to validate models threshold were moved and selected based on max of f1 score in P-R curves, being 0.1 in all models. To measure the validity of the models, the following measures were taken into account: accuracy, sensitivity, specificity, positive predictive value (PPV) and negative predictive value (NPV). Next, three different tests were applied to evaluate the performance of the model: area under the precision-recall curve (AUC-PR), Log Loss, and F1 score. These scores were selected because different studies recommend them for imbalanced data [22–24].”*

*References:*

*22.	Gaudreault JG, Branco P, Gama J. An Analysis of Performance Metrics for Imbalanced Classification. Lecture Notes in Computer Science (including subseries Lecture Notes in Artificial Intelligence and Lecture Notes in Bioinformatics) [Internet]. 2021 [cited 2023 Sep 15];12986 LNAI:67–77. Available from: https://link.springer.com/chapter/10.1007/978-3-030-88942-5_6*

*23.	Miao J, Zhu W. Precision–recall curve (PRC) classification trees. Evol Intell [Internet]. 2022 Sep 1 [cited 2023 Sep 15];15(3):1545–69. Available from: https://link.springer.com/article/10.1007/s12065-021-00565-2*

*24.	Bekkar M, Djemaa HK, Alitouche TA. Evaluation Measures for Models Assessment over Imbalanced Data Sets. Journal of Information Engineering and Applications [Internet]. 2013;3(10):27–38. Available from: http://www.iiste.org/Journals/index.php/JIEA/article/view/7633*

**12. The data normalisation at lines 259m to 261 should be placed in the methods section under data pre processing. I recommend that the authors rewrite the methods section and include a flowchart to facilitate comprehension.**

The normalization in these lines refers to the features importance scores. The method to compute scores for features importance was different for each algorithm and, because of that, the range of the scores differ among them. To facilitate the comparability of the features importance, we normalize these scores between 0 and 1 as in other studies. We have clarified it in lines 228-231:

*“After achieving valid and accurate models, the contribution of each variable to the prediction was extracted using caret R package. Methods applied for each algorithm are different and they give scores in different ranges so, to facilitate comparability, scores obtained for each method were normalized to a scale of 0–1.”*

*As suggested by the reviewer, we rewrite some parts of the methodology, specially the statistical analysis part and we divided it in 3 different sections.*

- *Machine learning models development (line 196)*
- *Machine learning models assessment (line 219)*
- *Variables importance (line 227)*

*Furthermore, in the flowchart, Figure 1, some specifications of the model development*

*process were depicted.*

Line 197:

“*Supervised machine learning algorithms were used to determine the utility of different variables to predict CVE. The process is depicted in Figure 1.”*

**13. AUC, not Accuracy, should be the primary performance metric for evaluating medical dataset models on lines 267 to 274. Clarify the AUC PR and F1-score. Please report the AUC value for tables 3 and 4, as it should be used to compare models. Include a comparison to the FRS score as well. Compare with other ML papers on CVD risk for the purpose of benchmarking.**

*As suggested by the reviewer and following different recommendations^[10]^^[11]^ for evaluation measures in studies with imbalanced data, we changed these lines (now lines 282-287) as follows:*

*“Table 4 compares the different measures used to evaluate model validity and performance. In terms of F1-score, the best results were obtained for the RF method (0.84). The only parameter for which the XGBoost method outperformed the RF method was specificity (53.00% and 52.05%, respectively). AUC-PR, Log Loss, and F1-Score indicated that the RF method performed best, while the NB method performed the worst (lowest AUC-PR and F1-Score and highest Log Loss).”*

*To clarify the AUC PR and F1-score we added the following sentence and add some references in lines 225-226:*

*“These scores were selected because different studies recommend them for imbalanced data [22–25].”*

*References:*

*22.	Miao J, Zhu W. Precision–recall curve (PRC) classification trees. Evol Intell [Internet]. 2022 Sep 1 [cited 2023 Sep 15];15(3):1545–69. Available from: https://link.springer.com/article/10.1007/s12065-021-00565-2*

*24.	Bekkar M, Djemaa HK, Alitouche TA. Evaluation Measures for Models Assessment over Imbalanced Data Sets. Journal of Information Engineering and Applications [Internet]. 2013;3(10):27–38. Available from: http://www.iiste.org/Journals/index.php/JIEA/article/view/7633*

*25.	Jiaming Yuan. Package ‘xgboost’. 2023 [cited 2023 Sep 14]; Available from: https://github.com/dmlc/xgboost/issues*

*Area under the curve for PR is shown in tables 3 and 4 (now tables 4 and 5) in 7^th^ column:*

Table 4: Evaluation of model validity and performance using only cardiovascular risk factors as predictive variables

| *Model* | *Accuracy (%)* | *Sensitivity (%)* | *Specificity (%)* | *PPV (%)* | *NPV (%)* | *AUC-PR* | *Log-loss* | *F1-Score* |
| --- | --- | --- | --- | --- | --- | --- | --- | --- |
| *XGBoost* | *71.29* | *72.71* | *53.00* | *95.20* | *13.16* | *0.15* | *0.24* | *0.83* |
| *Random Forest* | *73.96* | *75.66* | *52.05* | *95.29* | *14.30* | *0.17* | *0.24* | *0.84* |
| *Naïve bayes* | *68.29* | *69.96* | *47.00* | *94.42* | *10.88* | *0.11* | *0.26* | *0.80* |
|  | *PPV, positive predictable value; NPV, negative predictable value; AUC-PR, Area under the precision-recall curve.* | | | | | | | |

Table 5: Evaluation of model validity and performance using both cardiovascular risk factors and treatment exposure as predictive variables.

| *Model* | *Accuracy (%)* | *Sensitivity (%)* | *Specificity (%)* | *PPV (%)* | *NPV (%)* | *AUC-PR* | *Log-loss* | *F1-Score* |
| --- | --- | --- | --- | --- | --- | --- | --- | --- |
| *XGBoost* | *72.39* | *73.17* | *63.36* | *95.85* | *16.94* | *0.24* | *0.25* | *0.83* |
| *Random forest* | *73.35* | *73.68* | *69.52* | *96.55* | *18.57* | *0.28* | *0.24* | *0.84* |
| *Naïve bayes* | *61.78* | *61.88* | *60.62* | *94.79* | *12.07* | *0.13* | *0.28* | *0.75* |
|  | *PPV, positive predictable value; NPV, negative predictable value; AUC-PR, Area under the precision-recall curve.* | | | | | | | |

*As explained in comment 7, we did not calculate FRS in our study because we aimed to introduce medication adherence in our study and because some variables were not available in our cohort in the follow-up period.*

**14. I recommend that the authors implement variable importance, which can be generated using RF and XGB, in order to determine which features are of greater importance. Compare the results of the ML model to those of statistical analysis.**

*Thank you for your comment, as explained in previous commentaries, the feature analysis was conducted using caret package. To clarify it we have added one section in the methodology about variable importance and we expand the explanation of how it was done as follows (lines 227-243)*

*“After achieving valid and accurate models, the contribution of each variable to the prediction was extracted using caret R package. Methods applied for each algorithm are different and they give scores in different ranges so, to facilitate comparability, scores obtained for each method were normalized to a scale of 0–1. For RF models, the method applied to compute the contribution of each variable consisted of recoding for each tree the prediction accuracy on the out-of-bag portion. Then each predictor variable was permuted and the same was done. Finally, for all trees, the difference between both accuracies was averaged and normalized by the standard score [10,11].*

*For XG Boost models, the reduction in the loss function attributable to each variable in sum of squared error in predicting the gradient on each iteration was calculated. Finally, the improvement score for each predictor was averaged across all the trees in the ensemble [11,25].*

*Finally, for models developed applying NB a ROC curve analysis was conducted on each predictor. Different cutoffs were applied to the predictor data to predict the class. Then, sensitivity and specificity were computed for each cutoff and the area under ROC curve was calculated using the trapezoidal rule. This area was used as the measure of variable importance[11].”*

*References:*

*10.	Greenwell BM, Boehmke BC. Variable Importance Plots-An Introduction to the vip Package.*

*11.	Kuhn M. Building Predictive Models in R Using the caret Package. J Stat Softw [Internet]. 2008;28(5). Available from:* [*http://www.jstatsoft.org/*](http://www.jstatsoft.org/)

*12.	Wang K, Tian J, Zheng C, Yang H, Ren J, Liu Y, et al. Interpretable prediction of 3-year all-cause mortality in patients with heart failure caused by coronary heart disease based on machine learning and SHAP. Comput Biol Med. 2021 Oct 1;137:104813.*

*25.	Jiaming Yuan. Package ‘xgboost’. 2023 [cited 2023 Sep 14]; Available from: https://github.com/dmlc/xgboost/issues*

Reviewer #2: In the present work the authors have used several machine learning methods for the prediction of cardiovascular event incidence using two sets of features: cardiovascular risk factors and treatment exposure. They developed separate models and also combined models using these features and reported better prediction accuracies using combined feature set. I have few comments which are given beklow
Comments-
**1. The authors should mention how the predictive capacity for the features are being deduced?**

*Thank you for your comment. We analyse the importance of features applying caret package in R. To clarify it we added some lines in the methods as follows:*

*Methodology (lines 231-243):*

“*For RF models, the method applied to compute the contribution of each variable consisted of recoding for each tree the prediction accuracy on the out-of-bag portion. Then each predictor variable was permuted and the same was done. Finally, for all trees, the difference between both accuracies was averaged and normalized by the standard score [10,11].*

*For XG Boost models, the reduction in the loss function attributable to each variable in sum of squared error in predicting the gradient on each iteration was calculated. Finally, the improvement score for each predictor was averaged across all the trees in the ensemble [11,25].*

*Finally, for models developed applying NB a ROC curve analysis was conducted on each predictor. Different cutoffs were applied to the predictor data to predict the class. Then, sensitivity and specificity were computed for each cutoff and the area under ROC curve was calculated using the trapezoidal rule. This area was used as the measure of variable importance[11].”*

*References:*

*10.	Greenwell BM, Boehmke BC. Variable Importance Plots-An Introduction to the vip Package.*

*11.	Kuhn M. Building Predictive Models in R Using the caret Package. J Stat Softw [Internet]. 2008;28(5). Available from:* [*http://www.jstatsoft.org/*](http://www.jstatsoft.org/)

*12.	Wang K, Tian J, Zheng C, Yang H, Ren J, Liu Y, et al. Interpretable prediction of 3-year all-cause mortality in patients with heart failure caused by coronary heart disease based on machine learning and SHAP. Comput Biol Med. 2021 Oct 1;137:104813.*

*25.	Jiaming Yuan. Package ‘xgboost’. 2023 [cited 2023 Sep 14]; Available from: https://github.com/dmlc/xgboost/issues*

**2. There are no p-values for the overweight and obese features.**

*Thank you for your comment, the variable related to overweight and obesity was physical status. It was divided in three different categories: normal weight, overweight and obese, and the p-value for this variable is already shown in the table as follows:*

Table 3: Descriptive variables stratified according to incidence of cardiovascular events

|  | No CVE | CVE | p-value |
| --- | --- | --- | --- |
| N | 3448 (92.05) | 298 (7.95) |  |
| Age* | 61.50 (4.82) | 62.90 (4.20) | <0.001 |
| HYPERTENSION | 2252 (65.60) | 213 (71.70) | 0.039 |
| HC | 2765 (80.30) | 264 (89.2) | <0.001 |
| Diabetes | 567 (16.50) | 65 (22.00) | 0.019 |
| Physical status |  |  | 0.297 |
| Overweight | 1854 (54.50) | 157 (53.40) |  |
| Obese | 1009 (29.70) | 98 (33.30) |  |
| treatment exposure |  |  | <0.001 |
| Fully exposed | 1075 (45.60) | 51 (24.30) |  |
| Non-exposed | 485 (20.60) | 74 (35.20) |  |
| Partially exposed | 798 (33.80) | 85 (40.50) |  |
| CVE, cardiovascular event; HC, hypercholesterolemia. Data are expressed as the number (%); *In this case, as mean (SD). | | | |

1. Kuhn M. Building Predictive Models in R Using the caret Package. J Stat Softw . 2008;28(5).
2. Haibo He, Yunqian Ma. Imbalanced Learning: Foundations, Algorithms, and Applications. 1st Edition. Wiley-IEEE Press; 2013.
3. Maloof MA. Learning When Data Sets are Imbalanced and When Costs are Unequal and Unknown.
4. Collell G, Prelec D, Patil KR. Reviving Threshold-Moving: a Simple Plug-in Bagging Ensemble for Binary and Multiclass Imbalanced Data.
5. Miao J, Zhu W. Precision–recall curve (PRC) classification trees. Evol Intell. 2022;15(3):1545–69. Available from: https://link.springer.com/article/10.1007/s12065-021-00565-2
6. Marrugat J, Solanas P, D’Agostino R, Sullivan L, Ordovas J, Cordón F, et al. Estimación del riesgo coronario en España mediante la;56(3):253–61.
7. Ruiz-Villaverde G, Sánchez-Cano D, Ruiz-Villaverde R, Ábalos-Medina GM, Ramírez-Rodrigo J, Villaverde-Gutiérrez C. Agreement between Framingham-DORICA and SCORE scales in estimation of cardiovascular risk in the patients suffering from metabolic syndrome in Granada (Spain). Ir J Med Sci. 2011 Jun;180(2):351–4.
8. Amor AJ, Serra-Mir M, Martínez-González MA, Corella D, Salas-Salvadó J, Fitó M, et al. Prediction of Cardiovascular Disease by the Framingham-REGICOR Equation in the High-Risk PREDIMED Cohort: Impact of the Mediterranean Diet Across Different Risk Strata. J Am Heart Assoc. 2017;6(3).
9. Buitrago F, Calvo-Hueros JI, Cañón-Barroso L, Pozuelos-Estrada G, Molina-Martínez L, Espigares-Arroyo M, et al. Original and REGICOR Framingham functions in a nondiabetic population of a Spanish health care center: a validation study. Ann Fam Med. 2011;9(5):431–8.
10. Gaudreault JG, Branco P, Gama J. An Analysis of Performance Metrics for Imbalanced Classification. Lecture Notes in Computer Science (including subseries Lecture Notes in Artificial Intelligence and Lecture Notes in Bioinformatics). 2021;12986 LNAI:67–77. 6
11. Miao J, Zhu W. Precision–recall curve (PRC) classification trees. Evol Intell. 2022 Sep 1;15(3):1545–69.
