# Supplementary material for: Influence of cardiovascular risk factors and treatment exposure on cardiovascular event incidence: Assessment using machine learning algorithms
Source: PLoS One. 2023 Nov 16;18(11):e0293759. doi: 10.1371/journal.pone.0293759 (PMC10653526; doi:10.1371/journal.pone.0293759)
Supplement: Attachment — Submitted filename: Response to Reviewers.docx [file pone.0293759.s002.docx]

**Reviewer #1:**

**Thank you for addressing my concerns; however, the authors of this study neglected to include explainable AI to explain the black-box nature of the best machine learning algorithm.**

*We are sorry we did not explain ourselves well in the comment on explainable AI.*

*To evaluate the importance of the variables, the reviewer suggested to use LIME or SHAP methods. These techniques were not considered as in the present study the importance of the variables to overcome the black box nature of the machine learning algorithms was extracted using the caret package. It was explained in comment 3 of the previous review and in the manuscript, it is explained in lines 68-69 in the Introduction and lines 232-244 of the methodology. In addition, the results of these analysis are explained in Figures 2 and 3 and in lines 280-282, 294-299 and 322-327 in the results section.*

**The authors should also include the performance of the best algorithm and the number of datasets used in this study in the abstract section.**

*As suggested by the reviewer, we added the following sentences in the manuscript (lines 30 and 39):*

*Line 30: “The population of the study consisted of 3746 males.”*

*Line 39: “According to the performance of the algorithms, the most accurate was Random Forest when treatment exposure was considered (F1 score 0.84), followed by XGBoost.”*
